# CXCL9 induces chemotaxis, chemorepulsion and endothelial barrier disruption through CXCR3-mediated activation of melanoma cells

**DOI:** 10.1038/sj.bjc.6606056

**Published:** 2010-12-21

**Authors:** S Amatschek, R Lucas, A Eger, M Pflueger, H Hundsberger, C Knoll, S Grosse-Kracht, W Schuett, F Koszik, D Maurer, C Wiesner

**Affiliations:** 1 Division of Immunology, Allergy and Infectious Diseases, Department of Dermatology, Medical University of Vienna, Vienna, Austria; 2 Medical and Pharmaceutical Biotechnology, University of Applied Sciences, Piaristengasse 1, Krems A-3500, Austria; 3 Vascular Biology Center, Medical College of Georgia, 1459 Laney-Walker Blvd, Augusta, GA 30912-2500, USA

**Keywords:** CXCL9, fugetaxis, transendothelial migration, endothelial monolayer breakdown

## Abstract

**Background:**

Metastasis is associated with poor prognosis for melanoma. The formation of metastases is a multi-step process, in which cancer cells can subsequently acquire the potential to intravasate into the blood or lymph vessels, disseminate through the circulation, extravasate through the endothelium and invade the connective tissue. There is increasing evidence that chemokines have a pivotal role in the dissemination and establishment of melanoma metastasis.

**Methods:**

We isolated melanoma cells from melanoma metastasis and performed different migration assays and transendothelial resistance measurements of endothelial monolayers co-cultured with melanoma cells, in order to monitor barrier function and diapedesis and confirmed these results by confocal microscopy.

**Results:**

We observed that tumour endothelial cells (ECs) secrete high levels of CXCL9 in all, and CXCL10 in most melanoma metastases. Migration studies revealed that low concentrations of these chemokines induce chemotaxis, whereas high concentrations induce spontaneous migration of melanoma cells (chemokinesis/chemorepulsion) and the disruption of the endothelial barrier, resulting in an accelerated transendothelial migration (TEM). Addition of anti-CXCL9 or anti-CXCR3 antibodies to the co-cultures delayed the TEM of melanoma cells.

**Conclusion:**

Our data represent novel mechanisms by which tumour cells in melanoma metastases might use the chemokine-expressing endothelium to leave the tumour and eventually to form additional metastases at distinct sites.

Cancer cell invasion and metastasis are the primary causes of morbidity and mortality in human cancer. The development of malignant melanoma is a multi-step process including proliferation, differentiation, infiltration, intravasation into the lymph or bloodstream, survival, extravasation, invasion as well as adhesion and proliferation in distinct organ microenvironments (Hanahan and Weinberg, 2000). One of the rate-limiting steps in metastasis formation is the transendothelial migration (TEM) of the melanoma cells, which involves their adhesion to and subsequent transmigration through the endothelial cell (EC) monolayer (Sandig *et al*, 1997; Voura *et al*, 1998, 2001; Weis *et al*, 2004; Qi *et al*, 2005). Besides specific adhesion molecules, soluble factors such as growth factors, cytokines and chemokines are necessary to facilitate the extravasation process of the melanoma cells (Li and Zhu, 1999; Simiantonaki *et al*, 2002; Ramjeesingh *et al*, 2003).

Chemokines are small-protein signalling molecules that have a versatile and controversial role in tumour formation. Many chemokines show an antitumour effect by attracting immune cells to the tumour or by anti-angiogenic activity. By contrast, other chemokines may enhance tumour growth by acting as angiogenic factors, by stimulating proliferation of the tumour cells or by attracting tumour cells to a specific site (Rollins, 1997; Müller *et al*, 2001; Robledo *et al*, 2001; Wiley *et al*, 2001; Kawada *et al*, 2004). Chemokines that are found in the tumour environment, produced mainly by activated macrophages, are the CXCR3 ligands CXCL9/MIG and CXCL10/IP-10, attracting CXCR3-expressing T-cells and natural killer cells (Loetscher *et al*, 1996; Kunz *et al*, 1999; Romagnani *et al*, 2001a, 2001b; Payne and Cornelius, 2002). Numerous other chemokines, such as CXCL8 or CCL5, and chemokine receptors, such as CXCR4, CCR10 or CCR7, have been demonstrated to be expressed by melanoma cells (Payne and Cornelius, 2002).

In this study, we observed that the chemokine CXCL9 is highly secreted by tumour endothelial cells (TuECs) in melanoma metastases and that CXCR3 receptors are expressed on all isolated melanoma cells, regardless the tissue from which the metastasis was surgically removed. Low concentrations of the chemokine CXCL9 have a chemoattractive effect, whereas high concentrations have a repulsive effect on the melanoma cells. Stimulation with CXCL9 leads to spontaneous activation and consequently migration of the melanoma cells through an endothelial monolayer. Furthermore, pretreatment with CXCL9 increases the disruption of endothelial cell–cell contacts when the EC monolayer is brought in close contact with the melanoma cells.

## Materials and Methods

### Biochemicals and antibodies

Soluble human CXCL9 was purchased from PreproTech Inc., Rocky Hill, NJ, USA. Purified non-labelled mouse monoclonal antibodies were anti-CXCL9, anti-CXCR3, anti-CD31, (R&D Systems, McKinley Place, MN, USA) and Isotype IgG (Sigma-Aldrich, St Louis, MO, USA). FITC-conjugated anti-CXCR3 mAb and biotinylated goat IgG anti-human CD144 were purchased from R&D Systems, PE-conjugated anti-CD34 from BD Biosciences (San Jose, CA, USA) and PC5-conjugated anti-CD144 from Immunotech (Marseille, France). Lymphatic vessel-specific anti-podoplanin Ab was generated as described (Kriehuber *et al*, 2001).

### Cell culture

Human lymphatic endothelial cells (LECs) were obtained from ATCC (Manassas, VA, USA) and immortalised by ectopic expression of telomerase reverse transcriptase. Human umbilical vein endothelial cells (HUVECs) were obtained from PromoCell (Mannheim, Germany).

### Isolation of melanoma cells from metastatic melanoma

Metastatic melanoma (Supplementary Table I) with clear morphological discrimination to surrounding tissue was homogenised by cutting and treated with dispase (Invitrogen Life Technologies, Carlsbad, CA, USA) for 20 min at 37°C. Cells were seeded into fibronectin-coated (10 *μ*g ml^−1^; Sigma, Taufkirchen, Germany) wells and cultured in RPMI-1640 medium with 10% FCS, antibiotics and glutamine.

### Isolation of TuECs

Melanoma metastases were finely minced and incubated in Collagenase IV (0.5 U ml^−1^, Worthington, Freehold, NJ, USA) for 90 min at 37°C. After vigorous agitation, cells were pressed through a cell dissociation sieve (Sigma-Aldrich), accompanied by extensive washing with cold RPMI (10% FCS). Cells were centrifuged and stained with anti-CD144 for immunomagnetic enrichment of ECs using anti-IgG1 immunomagnetic beads (Miltenyi Biotec, Auburn, CA, USA). Enriched cell suspensions were incubated with PE-conjugated anti-CD34 and anti-podoplanin (anti-rabbit Alexa-488; Molecular Probes, Eugene, OR, USA). Cells were sorted using the FACSAria (BD Biosciences) to a purity of at least 98%.

### Isolation of ECs from normal skin

The skin was obtained from healthy adults undergoing elective plastic surgery. Dermal sheets were prepared by incubation of split-thickness skin (0.8 mm) with DispaseI (3 U ml^−1^; Roche, Mannheim, Germany) for 60 min at 37°C, and subsequent removal of the epidermis. Endothelial cells were released from the tissue by scraping, enriched and sorted as described above.

All human tissue specimens were obtained upon informed consent. All samples of melanoma metastases were collected at the General Hospital Vienna following approval by an Institutional Review Board and written informed consent (patient ID: N, normal skin, T, tumour metastases).

### Quantitative real-time PCR

Total RNA was extracted using the High Pure RNA Isolation Kit (Roche). RNA was reverse transcribed with the TaqMan 5′ nuclease RT–PCR assay (Applied Biosystems, Foster City, CA, USA). TaqMan primer and probe sequences for B2M (internal control), CXCL9 and CXCL10 were purchased commercially (Applied Biosystems). Reactions were run on a spectrofluorometric thermal cycler (MX 4000; Stratagene, La Jolla, CA, USA). Threshold cycle (*C*_t_) values of the target genes were converted to arbitrary expression values by extrapolation from the standard curve and finally normalised to the internal control.

### Cloning of CXCL9 and expression vectors

RNA was isolated from interferon-*γ* (Invitrogen, Life Technologies)-stimulated HUVECs using the RNeasy kit, (Qiagen, Hilden, Germany). After DNaseI treatment, 1 *μ*g total RNA, oligo(dT)_15_ pimer and reverse transcriptase (Roche) was used for RT–PCR and CXCL9: forward primer, 5′-aggagtgacttggaactccatt-3′ reverse primer, 5′-tggggacaagatgagaaagg-3′ for PCR reaction (MyCycler, Bio-Rad, Richmond, CA, USA). CXCL9 DNA was cloned into pIRES-EGFP vector by blunt end cloning.

### Cell transfections

Endothelial cells (4 × 10^5^) per well were transfected by the lipofectamin-2000 method using 2 *μ*g DNA and 7 *μ*l lipofectamin (Invitrogen). Transfected cells were rinsed 4 h later and incubated for further 16 h before they were used for TEM assay.

### Transwell/transendothelial/migration assays

Chemotaxis assays were performed as described previously (Charbonnier *et al*, 1999), except that 96-well Transwell plates (Costar, Cambridge, MA, USA) with 8 *μ*m pore size were used and migrated melanoma cells were collected and measured by Multiplate Reader (Paradigm, Beckman Coulter, Fullerton, CA, USA). Different concentrations of the chemokine solutions or buffer alone were added to the upper (chemorepulsion) or to the lower chamber (chemotaxis) to individual wells or to both chambers for checkerboard analysis. For the TEM assay, the filters were pre-coated with 10 *μ*g ml^−1^ fibronectin (Sigma) or 1% gelatine (Sigma) on the upper site. Endothelial cell-monolayers and melanoma cells were cultivated in medium with 1% FCS with or without CXCL9 for 8 h before melanoma cells (Cell Tracker-568) were allowed to migrate for 8 or 14 h.

Chemokinesis assay was performed by using the Oris Cell Migration Assay from Platypus Technologies (Madison, WI, USA), utilises cell seeding stoppers to create a pristine, 2 mm diameter detection zone in the centre of each well. Melanoma-7 cells (30 000) were dispensed into each well of a 96-well microplate populated with the cell seeding stoppers, and incubated for 24 h before the stoppers were removed and cells either non-treated or treated with soluble chemokine CXCL9 in a concentration-dependent manner. After migration for 16 h, cells were stained with Cell Tracker-488 (Molecular Probes), and the Oris Detection Mask was applied to the bottom of the microplate. Cells that have migrated into the detection zone are imaged with the Multiplate Reader.

### Flow cytometric analysis

For FACScan, cells (2 to 4 × 10^5^) were either fixed and permeabilised (Fix & Perm Reagents, Invitrogen) or not fixed and resuspended in PBS, 0.1% BSA (Biological Industries, Kibbutz Beil, Haemek, Israel). Immunofluorescence was performed by exposing cells to antibodies (see Biochemicals and Antibodies) or Isotype IgG (Sigma-Aldrich) as negative control for 30 min on ice and to 1 *μ*g ml^−1^ propidium iodide (Sigma, 1 : 100) to gate out dead cells. After staining, cells were analysed using flow cytometry and CellQuest software (BD Biosciences).

### Electrical cell-substrate impedance sensing technology

Endothelial cell-monolayer breakdown (transmigration) assay was performed using the electrical cell-substrate impedance sensing (ECIS) model 1600R (Applied BioPhysics, Troy, NY, USA). The measurement system consists of an eight-well cell culture dish (8W10E plate) containing a small active electrode (5 × 10^−4^ cm^2^) and a large counter electrode (0.15 cm^2^) deposited on the bottom of each well. A small amplitude AC signal is imposed across the pair of electrodes, onto which cells are deposited. The resulting impedance is being calculated by the ECIS device (Wegener *et al*, 2000). For the EC monolayer breakdown assays, 2 × 10^5^ human LECs were grown to confluent monolayers and treated with different concentrations of CXCL9 with or without anti-CXCL9 or anti-CXCR3 antibody for 1 h. Melanoma cells (1 × 10^5^) were seeded on top of the EC-monolayers and EC breakdown was assessed by continuous resistance measurements for 8–10 h.

### Immunostaining and immunohistochemistry

Melanoma cells, either co-cultured with human ECs or alone were seeded into fibronectin-coated (10 *μ*g ml^−1^; Sigma) chamber slides. After 24 h, cells were fixed with 4% paraformaldehyde in PBS for 15 min and permeabilised with 0.1% Triton X-100 in PBS for 3 min. Proteins were immunodetected with 5 *μ*g ml^−1^ monoclonal anti-CXCR3 antibody (Alexa-488), 10 *μ*g ml^−1^ anti-CD144 (Alexa-633), and phalloidin-568 (Molecular Probes). Chamber slides were mounted onto coverslips in 60% glycerol-Tris-buffered saline, and viewed under a confocal microscope (LSM 510, Zeiss, Oberkochen, Germany). Five-micrometre cryosections of normal skin or metastatic melanoma were mounted onto glass slides, air-dried, and fixed with acetone for 20 min. After drying, slides were hydrated with Ca_2_/Mg_2_-deficient PBS and exposed to anti-CXCL9 antibody (Isotype-matched control antibodies), anti-CD144 following Alexa-488, Alexa-633 and phalloidin-568 exposure.

For the EC monolayer breakdown assay, 2 × 10^5^ HUVECs were grown to confluent monolayers in fibronectin-coated (10 *μ*g ml^−1^; Sigma) chamber slides and stimulated with 400 ng CXCL9 for 30 min. Melanoma-7 cells (3 × 10^5^) were seeded on the monolayer and endothelial breakdown was allowed for 6 h at 37°C. Chambers were fixed and stained with 3 *μ*g ml^−1^ anti-CD31 antibody.

### Statistical analysis

The Student's paired *t*-test was used, when the data were normally distributed. Reported *P*-values are two-tailed, and *P*<0.05 was considered statistically significant and *P*<0.01 was considered as statistically highly significant. The MFI values were obtained by subtracting the MFI of the isotype control from the MFI of the positively stained sample.

## Results

### Expression of chemokine receptors in freshly isolated metastatic human melanoma cells

**Figure 1:**
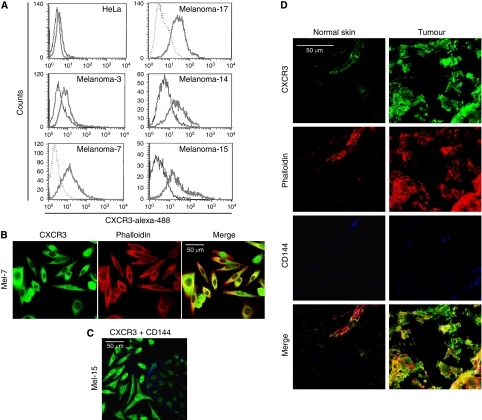
Expression of CXCR3 on human melanoma cells. (**A**) Human melanoma cells isolated from melanoma metastases were stained for surface CXCR3 and analysed by flow cytometry. Grey and bold histogram, CXCR3 staining; black or spotted histogram, isotype staining. (**B**) Melanoma-7 were immunostained with anti-CXCR3 antibody, Alexa488-conjugated anti-mouse as secondary reagent, and for actin with phalloidin-568 and viewed by confocal laser scanning microscopy; bar represents 50 *μ*m. (**C**) Melanoma-15 was co-cultured with HUVECs and stained with anti-CXCR3 (Alexa-488) and anti-CD144 (Alexa-633) antibodies; bar represents 50 *μ*m. (**D**) Immunolocalisation of CXCR3 in human skin and melanoma metastasis. Cryosections of human skin and melanoma metastasis were stained with anti-CXCR3 antibody (Alexa-488), anti-CD144 antibody (Alexa-633) and phalloidin-568.

Freshly isolated human melanoma cells, obtained from metastatic melanoma patients, were isolated and cultivated for a few passages or used directly (primary melanoma T14, T15) for binding of antimelanoma antibodies (Melanin A, Tyrosinase, HMB45). Positive cells were subsequently used to determine expression of various chemokine receptors (CCR1-CCR9 and CXCR1-CXCR6) in fixed and permeabilised melanoma cells by flow cytometry (Supplementary Tables I and II). As such, we could detect expressions of CCR5, CCR9 and CXCR3 in all malignant metastatic melanoma cells, using a immunohistochemistry protocol described previously (Robledo *et al*, 2001). Moreover, we confirmed the expression of the CXCR3 receptor on the surface of living melanoma cells by flow cytometry (Figure 1A). To study the cellular distribution of this receptor, melanoma cells (Mel-7 and Mel-15) were grown on fibronectin-coated chamber slides for 24 h, fixed, permeabilised and immunostained with anti-CXCR3 antibody (Alexa-488) and phalloidin-568 (Figure 1B). Alternatively, the melanoma cells were co-cultivated with human ECs, stained with anti-CXCR3 (Alexa-488) and anti-CD144 (Alexa-633) antibodies and subsequently investigated using confocal laser scanning microscopy (Figure 1C). Both flow cytometric and immunofluorescence microscopic analysis confirmed CXCR3 expression and demonstrated that the receptor is mainly localised in the cytoplasm, although it can also be found in the plasma membrane. Moreover, CXCR3 expression is higher in melanoma cells than in ECs (Figure 1C). To assess whether CXCR3 is also expressed by melanoma cells in metastatic tumour tissues, we performed immunofluorescence and confocal microscopy from normal skin and melanoma metastases, using anti-CXCR3 (Alexa-488) antibody, anti-CD144 (Alexa-633) antibody and phalloidin-568. Analysis of lymph node metastases revealed high expression of CXCR3 in melanoma cells (Figure 1D), which could also be observed in ECs isolated from both normal skin and tumour tissue. Taken together, these data demonstrate that human melanoma cells express the chemokine receptor CXCR3 not only within the tumour tissue, but also even after isolation and cultivation.

### Expression of CXCL9 in TuECs

**Figure 2:**
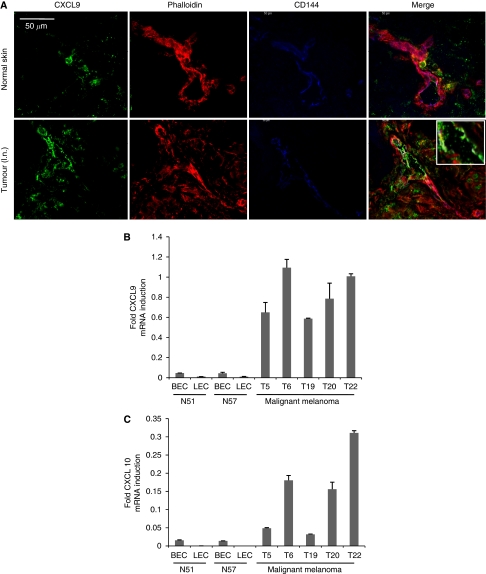
Expression of CXCR3 ligands, CXCL9 and CXCL10, in human tumour endothelial cells. (**A**) Immunolocalisation of CXCL9 in human skin and melanoma metastases. Cryosections of normal human skin and melanoma metastasis (lymph node) were stained with anti-CXCL9 antibody (Alexa-488), anti-CD144 antibody (Alexa-568), and phalloidin-568 and viewed by confocal laser scanning microscopy; bar represents 50 *μ*m. Insert indicates tumour vessel in a higher magnification (dotted structure). (**B**, **C**) Quantitative Real-time PCR of CXCL9 and CXCL10. Blood ECs, LECs and TuECs were isolated from human skin and melanoma metastasis by cell sorting using anti-CD34, anti-CD144 and anti-podoplanin antibodies, total RNA was isolated, reverse transcribed and corresponding cDNA subjected to TaqMan PCR using commercial probes and primers for CXCL9 and CXCL10. All data shown are mean values and standard error of means from two independent PCR measurements, and have been normalised to the internal control gene B2M (N, normal skin; T, malignant melanoma).

As our previous results had shown a prominent expression of the CXCR3 chemokine receptor in human melanoma metastases, we subsequently wanted to determine whether also the CXCR3 ligands are expressed in melanoma metastases, by means of performing immunofluorescence and confocal microscopy of melanoma cells isolated from normal skin and melanoma metastases, using anti-CXCL9 (Alexa-488) antibody, anti-CD144 (Alexa-633) antibody and phalloidin-568. As such, we observed that CXCL9 is highly expressed in TuECs, especially in their luminal (apical) side of the tumour vessel (Figure 2A). To further confirm that TuECs express high levels of CXCR3 ligands, we isolated LECs and blood endothelial cells (BECs) from normal human skin and TuECs from melanoma metastases and compared the expression levels of CXCL9 and CXCL10 using RT–PCR. We could not detect podoplanin-positive ECs (lymphatic TuECs) in suspensions of melanoma lesions, although this population made up 40–60% of total ECs in normal skin (Supplementary Figure 2). Consequently, only a single population of TuECs was isolated from tumour samples. Tumour endothelial cells demonstrated a more than 11-fold increase in CXCL9 mRNA expression and a 3- to 20-fold increase in CXCL10 mRNA expression, as compared with BECs or LECs in all samples (Figure 2B and C). Samples used were isolated from patients who did not receive IFN-*α* or IFN-*γ* treatment for at least 1 month. Taken together, these results show that TuECs express high levels of the CXCR3 ligands CXCL9 and CXCL10.

### The CXCR3 ligand CXCL9 can induce spontaneous melanoma cell migration

**Figure 3:**
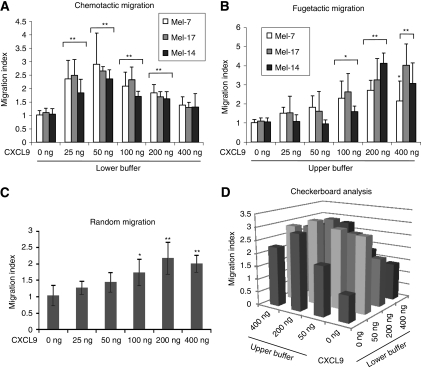
CXCL9 induces chemoattraction, chemorepulsion and chemokinesis in melanoma cells. Melanoma cells were assayed for chemoattraction and chemorepulsion in response to CXCL9 added either to the lower (chemoattraction) or to the upper (chemorepulsion) chamber of the 96-well Transwells. (**A**) Chemotactic response of Mel-7, -17 and -14 towards (chemoattraction) soluble CXCL9 or away (chemorepulsion) the soluble chemokine CXCL9 (**B**) was probed in the concentrations 0–400 ng ml^−1^. (**C**) Melanoma-7 were seeded on 96-well plate populated with cell seeding stoppers. After 24 h, the cells adhered to the surface around the stopper tips. Stoppers were removed and melanoma cells were treated with 0–400 ng ml^−1^ soluble CXCL9 and allowed to migrate (chemokinesis) into the empty space for 16 h before cells were stained with Cell Tracker-488, and cells that have migrated into the detection zone are imaged using the Detection Mask and the Multiplate Reader. (**D**) Mel-7 cells were assayed for haptotaxis, chemotaxis and chemorepulsion in response to CXCL9 added to the upper and to the lower chamber of the 96-well Transwells. Mean migration indices (+s.d., *n*⩾5 experiments) are given on the *Y* axis (^*^P<0.05; ^**^P<0.01, Student's *t*-test).

**Figure 4:**
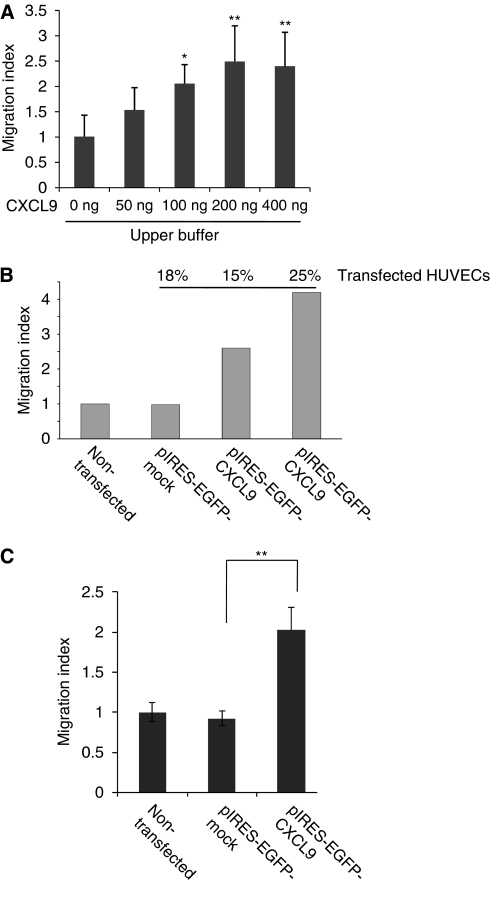
Soluble CXCL9 and CXCL9-expressing ECs increase melanoma cells migration through an EC monolayer. (**A**) Melanoma-7 cells (Cell Tracker-568) were seeded in the upper chamber and allowed to migrate through the LEC monolayer and 8-*μ*m pore-size filters for 8 h and were measured by Multiplate Reader. (**B**) Human umbilical vein endothelial cells (HUVECs) were transfected with CXCL9 expression constructs (pIRES-EGFP-CXCL9), mock transfected or non-transfected and seeded on fibronectin-coated Transwell chambers for 24 h (monolayer formation). Mel-7 cells were seeded in the upper chamber and allowed to attach and migrate through the HUVEC monolayer and 8-*μ*m pore-size filters for 8 h. The percentage of transfected HUVECs were defined by FACS (EGFP-positive cells) analysis. (**C**) Human LECs were transfected (20% transfection efficiency) as described in **B** and seeded on gelatine-coated Transwell chambers for 24 h before Mel-7 cells (Cell tracker-568) were allowed to attach and migrate through the EC monolayer for 14 h. Migrated melanoma cells in the lower part of the filter and in the fluid phase of the lower chamber were gathered and relative fluorescence detected by Multiplate Reader (^*^P<0.05; ^**^P<0.01, Student's *t*-test).

To better understand the relevance of the overexpression of the chemokine CXCL9 in TuECs, we have examined in the following experiments several migratory steps deemed to be important for metastasis, including migration, endothelial cell–cell breakdown and TEM. To determine whether CXCL9 can induce chemoattraction of melanoma cells, three different melanoma cell lines were stained with Cell Tracker-568, resuspended in migration buffer and placed into the upper chamber of 96-well Transwells. The lower chamber of the Transwells contained various concentrations (0–400 ng ml^−1^) of the chemokine CXCL9 in the buffer (Figure 3A). Migration was allowed for 8 h, after which cells in the lower part of the filter were collected and measured in a Multiplate Reader. As such, we found that CXCL9 is able to trigger a chemotactic response on all analysed cell lines, with the highest migration detected at 50 ng ml^−1^ soluble CXCL9, followed by 25 and 100 ng ml^−1^ (Figure 4A). These results are consistent with the ones from previous studies (Robledo *et al*, 2001; Kawada *et al*, 2004).

To investigate whether melanoma cells can also be repelled by this chemokine, Transwell assays were performed as described above, except that the soluble chemokine was added to the upper buffer (Figure 3B). As such, we detected that 200 and 400 ng ml^−1^ of soluble CXCL9, when added to the upper chamber, resulted in a highly significant fugetactic migration of Melanoma-7 (200 ng ml^−1^: 2.71±0.52; 400 ng ml^−1^: 2.16±1.03), Melanoma-17 (200 ng ml^−1^: 3.25±1.10; 400 ng ml^−1^: 4.08±1.12) and Melanoma-14 cells (200 ng ml^−1^: 4.12±0.53; 400 ng ml^−1^ 3.06±1.08). Soluble CXCL10 at concentrations of 200 and 400 ng ml^−1^ had a tendency to induce chemorepulsive migration, albeit not significant (data not shown). None of the treatments with 0–400 ng ml^−1^ CXCL9 or CXCL10 influenced cell death or cell proliferation in melanoma cells (data not shown).

To examine whether the melanoma cells migrate directionally (along or away a chemogradient) or randomly (chemokinesis), the Oris Cell Migration Assay was preformed, as described in Materials and Methods. Melanoma-7 cells were plated around cell seeding stoppers for 24 h before stoppers were removed. Cells (labelled by Cell Tracker-488) were treated with 0–400 ng ml^−1^ CXCL9, and the degree of migration into the detection zone was assessed by measuring the fluorescence intensity after 16 h (Figure 3C). CXCL9 revealed a dose-dependent increase of fluorescence (migrated melanoma cells) in the detection zone. The highest chemokinesis was detected when Mel-7 cells were stimulated with 200 ng ml^−1^ soluble CXCL9 (2.13±0.48) followed by 400 ng ml^−1^ (1.98±0.29). To further discriminate between haptotaxis, chemotaxis and chemorepulsion, checkerboard analysis was performed by adding various concentrations of the soluble chemokine to the upper and to the lower buffer to individual wells of the Transwell system (Figure 3D). The highest Mel-7 migration was detected when 200 ng ml^−1^ of soluble CXCL9 was added in the upper chamber, and 50 ng ml^−1^ (200/50 ng ml^−1^) in the lower chamber (3.2±0.31), followed by 200/200 ng /ml^−1^ (3.1±0.41) and 50/50 ng (2.9±0.57). These data confirm that CXCL9 promotes random melanoma cell migration with the tendency of the melanoma cells to migrate away from the chemogradient (chemorepulsion) at high concentrations.

### Soluble CXCL9 and CXCL9-expressing ECs increase TEM of melanoma cells

To further determine whether the activating effect of CXCL9 promotes TEM of melanoma cells, HUVECs were cultivated on fibronectin-coated 8 *μ*m filters of a Transwell plate until reaching confluence, after which the assay was performed as described above (Figure 4A). Migration was allowed for 16 h, after which melanoma cells were collected and measured in the Multiplate Reader. The chemotactic repulsion and the subsequent TEM were stimulated by soluble CXCL9 in a dose-dependent manner (Figure 4A). Under these conditions, the highest migration through the EC monolayer could be found at 200 ng ml^−1^ soluble CXCL9. The highest chemotactic migration and subsequent TEM were found at 100 ng ml^−1^ soluble CXCL9 (data not shown). These data indicate that CXCL9 not only promotes chemotaxis towards, but also chemorepulsion or chemokinesis away from a chemogradient, leading to the subsequent migration of melanoma cells through an EC monolayer.

To determine whether CXCL9-expressing ECs promote the TEM of melanoma cells, HUVECs were either transfected with pIRES-EGFP-CXCL9 plasmid, mock transfected or non-transfected and subsequently seeded onto fibronectin-coated Transwell culture chambers until confluent monolayers were formed. Transmigration of Melanoma-7 cells (Cell Tracker-568) through monolayers with 15 or 25% CXCL9-transfected, 18% mock-transfected or non-transfected HUVECs (transfection efficiency defined by FACS analysis (EGFP-positive cells)) was allowed for 8 h. As shown in Figure 4B, CXCL9 expression in ECs increased the number of melanoma cells migrating through the HUVEC monolayer 2.5-fold when 15% ECs expressed CXCL9 or 4.2-fold when 25% of ECs expressed CXCL9, whereas mock transfection showed no effect. Similar results were obtained when human LECs (instead of HUVECs) were transfected with pIRES-EGFP-CXCL9 plasmid, mock-transfected or non-transfected and seeded onto gelatin (1%)-coated Transwell culture chambers. Transendothelial migration was enhanced two-fold if CXCL9 was expressed (Figure 4C). All the culture conditions were performed in quintuplicate. Almost all migrated melanoma cells have been found on the underside of the filter. Taken together, these results indicate that CXCL9 expression in ECs promotes TEM of melanoma cells.

### CXCL9 activates melanoma cell migration, resulting in an enhanced EC monolayer breakdown during melanoma infiltration

**Figure 5:**
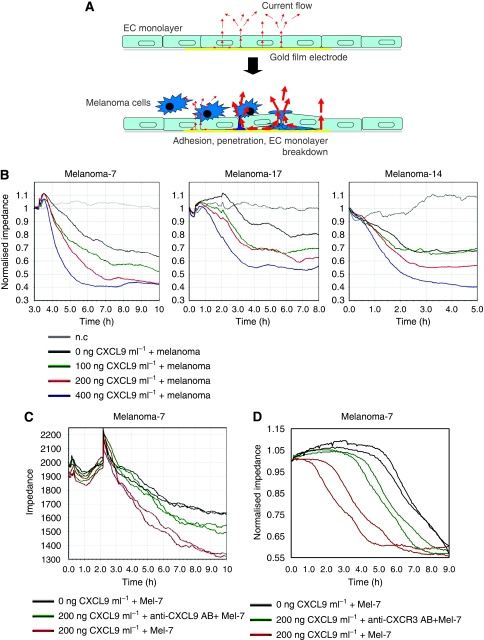
CXCL9 promotes EC monolayer disruption and melanoma cell invasion in a concentration-dependent manner. (**A**) Schematic diagram of the ECIS technology adapted for the EC monolayer disruption assay. (**B**) Lymphatic endothelial cells were seeded onto ECIS arrays and allowed to grow to a monolayer before treated with different concentrations of soluble CXCL9 or not treated and challenged with melanoma cells (Mel-7, -17, -14) 1 h later. Endothelial cell monolayer breakdown during melanoma cell transmigration was assessed by continuous resistance measurements for 5–10 h. (**C**) Same approach as in **B**, except that the 200 ng ml^−1^ CXCL9 was preincubated with anti-CXCL9 antibody for 1 h before the monolayer was overlaid with Mel-7 cells. (**D**) Same approach as in **C**, except that melanoma cells were preincubated with anti-CXCR3 antibody for 1 h.

**Figure 6:**
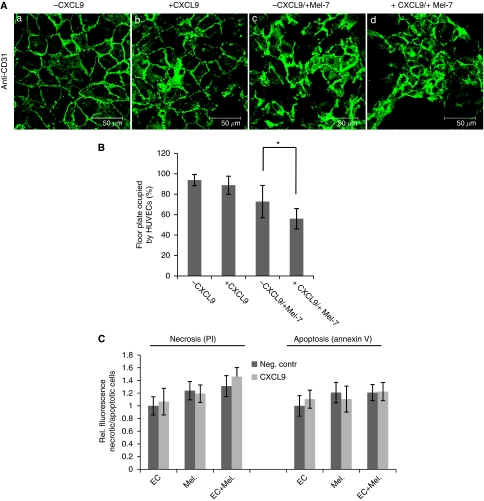
CXCL9 enhances cell–cell disruption during melanoma cell infiltration. (**A**) Human umbilical vein endothelial cell (HUVEC) monolayers were stimulated or not stimulated with CXCL9 for 30 min before being overlaid with Mel-7 cells. Attachment and endothelial breakdown were allowed for 6 h at 37°C before cells were fixed and stained with anti-CD31 antibody. (**B**) Percentage (%) of the floor plate occupied by HUVECs (+s.d., *n*⩾3 experiments and counts) was given on the *Y* axis. (**C**) Same approach as in **A**, except that apoptotic and necrotic cells were stained with Annexin V and PI, respectively (^*^P<0.05; Student's *t*-test).

To study the influence of CXCL9 expression on endothelial barrier function during melanoma cell attachment and infiltration, we used the ECIS technology (Figure 5A). Endothelial cell monolayer disruption can thus be monitored online, by means of measuring the decrease in impedance over time. As such, substances that influence the infiltration of cells into an EC monolayer can be detected, as they will lead to a changed resistance of the monolayer. Lymphatic endothelial cells were cultured onto ECIS arrays and formed a confluent monolayer within 24 h (Figure 5A–E). The monolayers were pretreated with 0, 100, 200 or 400 ng ml^−1^ soluble CXCL9, before being challenged with Mel-7, Mel-17 and Mel-14 cells or remaining unchallenged (Figure 5B, grey lines). Endothelial barrier function was subsequently assessed by continuous resistance measurements. CXCL9 increased the EC monolayer disruption during melanoma infiltration in all three primary melanoma cells in a concentration-dependent manner, (Figure 5B). No change in resistance could be detected when monolayers were stimulated with CXCL9 itself or with substances secreted by melanoma cells, contained in the supernatant (Figure 6Aa and b, Supplementary Figure 1A). When Mel-7 cells were pulsed for 1 h with 200 ng ml^−1^ soluble CXCL9 and subsequently washed before overlaying them onto the EC monolayer, EC breakdown was increased, in comparison with non-pulsed cells (Supplementary Figure 1B). Penetration of melanoma cells could be significantly decreased when CXCL9 was preincubated with anti-CXCL9 antibody (Figure 5C) or when the melanoma cells were treated with anti-CXCR3 antibody for 1 h (Figure 5D) before the challenge of the EC monolayer. These results show that CXCL9 promotes not only the migration (Figure 3), but also the penetration of melanoma cells through the EC monolayer, resulting in an enhanced breakdown of the latter.

### The soluble chemokine CXCL9 promotes cell–cell disruption in endothelial monolayers during TEM

To further investigate the influence of soluble CXCL9 during endothelial breakdown and to detect whether the melanoma cells were able to form holes, which could be due either by increased endothelial apoptosis/necrosis and/or by cell–cell contact disruption during transmigration in the endothelial monolayers, HUVECs were grown to confluence and were subsequently stimulated with 200 ng ml^−1^ soluble CXCL9. Thirty minutes later, the HUVEC monolayer was challenged with Mel-7 cells, after which endothelial breakdown was allowed to occur for 6 h, before cells were fixed and stained with anti-CD31 antibody (Figure 6A). When cells were preincubated with soluble CXCL9, melanoma cell infiltration led to increased hole formation and to complete monolayer disruption (Figure 6Ac and d). When measuring the fields that were still occupied with HUVECs, we found that stimulation with soluble CXCL9 increased the disruption of the monolayer after melanoma treatment, whereas CXCL9 treatment by itself had no effect (Figure 6B). To find out whether this effect is triggered by apoptosis, necrosis or simply by endothelial cell–cell contact disruption, a migration assay was performed as described above, in which cells were stained with AnnexinV/PI and measured in the Multiplate Reader (Figure 6C). Neither apoptosis nor necrosis could be detected, not even when the endothelial monolayer was preincubated with CXCL9. In agreement with Mierke *et al* (2008), these findings suggest that the melanoma cells infiltrated not by triggering apoptosis or necrosis in ECs, but rather by disrupting the endothelial cell–cell contacts.

## Discussion

Solid tumours are organ-like structures, containing both malignant tumour and stroma cells, which contain extracellular matrix and different extracellular molecules, such as growth factors, adhesion molecules and chemokines. All of these components in the tumour stroma have a strong influence on tumour cell proliferation, invasion and metastasis (Aznavoorian *et al*, 1990; Ohtani, 1998; Balkwill and Mantovani, 2001; Bissell and Radisky, 2001). Chemokines and chemokine receptors, secreted either by the tumour cells themselves or by stroma cells, are often strongly upregulated during tumourigenesis, can lead to increased angiogenesis and tumour growth, to the stimulation of the immune response to the tumour and to the facilitation of metastasis (Wang *et al*, 1998; Payne and Cornelius, 2002; Kakinuma and Hwang, 2006; Raman *et al*, 2007). In this report, we have demonstrated that the chemokines CXCL9 and CXCL10 are highly expressed in TuECs. We also found that the receptor for these ligands, that is, the CXCR3 protein, was expressed in all isolated, living melanoma cells. CXCR3 expression in human and murine melanoma cell lines has been suggested by others to mediate directional cell migration along the chemokine gradients of CXCL9, CXCL10 and CXCL11 (Robledo *et al*, 2001; Kawada *et al*, 2004). The results presented in this study demonstrate that CXCL9 secreted by TuECs triggers different types of directional migration, that is, chemotaxis, fugetaxis and chemokinesis in melanoma cells in a concentration-dependent manner. This chemorepellent signal has been described first by Vianello *et al* (2005), who reported that mature single-positive CD4 cells emigrate from the fetal thymus on treatment with high concentrations of CXCL12/SDF-1, by means of concentration-dependent and CXCR4 receptor-mediated fugetaxis (Vianello *et al*, 2005, 2006). In this report, we show for the first time that high concentrations of the soluble chemokine CXCL9 preferentially promote chemokinesis and the chemorepulsive migration of human melanoma cells rather than the movement towards the chemogradient (Figure 3).

In our *in vitro* studies reported here we could further demonstrate that soluble CXCL9 promotes the migration of melanoma cells through an EC monolayer in a dose-dependent manner. Although the chemokines CXCL9, CXCL10 and CXCL11 were reported to have an angiostatic effect when expressed by melanoma cells (Romagnani *et al*, 2001a, 2001b; Mehrad *et al*, 2007; Keeley *et al*, 2008), our study suggests that the chemokine CXCL9 may promote additional metastasis, when it is expressed in TuECs, by means of increasing transendothelial and melanoma migration. In this report, we demonstrate that the CXCL9/CXCR3 interaction exhibits a strong chemotactic efficacy for melanoma cells, not only in chemotactic migration but also in fugetaxis as well as in TEM, processes that are crucial for the dissemination of melanoma cells and for the further establishment of novel melanoma metastases.

It was suggested that in tumours the cancer cells are often integrated into the vessel wall, making tumour vessels leaky or causing complete disruption of the endothelial barrier (Stewart *et al*, 1987; Hashizume *et al*, 2000; Heyder *et al*, 2002). We used the sensitive and reproducible ECIS technology to monitor endothelial monolayer integrity during melanoma cell infiltration in real time, and measured the influence of different soluble CXCL9 concentrations. As expected from the TEM assays, the addition of soluble CXCL9 significantly accelerated melanoma-mediated breakdown of the EC monolayer in a concentration-dependent manner. In addition, we were able to confirm that this increased EC monolayer breakdown was not mediated by binding of CXCL9 ligand to the CXCR3 receptor presented by ECs, but due to the ligand/receptor interaction on melanoma cells. This was also confirmed by the fact that the monolayer breakdown was blunted when the soluble chemokine was neutralised with anti-CXCL9 antibody or when the melanoma cells were pretreated with anti-CXCR3 antibody.

These findings suggest that CXCL9-activated melanoma cells reinforce the leakiness of the tumour vessels, which may lead to increased intravasation and dissemination of the melanoma cells. Immunostaining experiments revealed that upon CXCL9 stimulation, melanoma transmigration led to the formation of ‘holes’ in the endothelial monolayer and subsequently to the complete EC monolayer disruption. In contrast to several reports, we found no indication of triggering of the ECs towards apoptosis or necrosis during the TEM of the melanoma cells, suggesting that the breakdown of the endothelial monolayer is caused by the disruption of the cell–cell contacts (Li *et al*, 2001; Heyder *et al*, 2002; Lasagni *et al*, 2003; Mierke *et al*, 2008).

As a result of our study, one could argue that CXCL9 expressed by tumour stroma cells promotes the escape of the melanoma cells from tumour metastases. On the one hand, the chemokine CXCL9 has been described as a key mediator in homing and metastasis, as confirmed by the fact that expression of CXCL9 and CXCL10 in the lymph nodes facilitates melanoma cell metastasis in a murine model (Kawada *et al*, 2004). By contrast, however, it was demonstrated that the application of chemokines and cytokines, either together with chemotherapy or as an alternative, can be beneficial for the treatment of malignancies in experimental animal models (Sgadari *et al*, 1997; Kanegane *et al*, 1998; Rodolfo and Colombo, 1999; Clinton *et al*, 2000; Keilholz and Eggermont, 2000). Owing to the fact that the treatment of melanoma metastases with interferon-*α* or interferon-*γ* alone or in combination with dacarbazine (or other chemotherapeutic) shows only little or no significant difference in median survival or overall response rate (Shepherd and Milne, 2000), it might be possible that the melanoma cells overcome the antitumour effect by exploiting the high CXCL9 expression to escape from the tumour and to form novel metastases at distant sites.
