# Supplementary material for: CXCL9 induces chemotaxis, chemorepulsion and endothelial barrier disruption through CXCR3-mediated activation of melanoma cells
Source: Br J Cancer. 2010 Dec 21;104(3):469–79. doi: 10.1038/sj.bjc.6606056 (PMC3049560; doi:10.1038/sj.bjc.6606056)
Supplement: Supplementary Tables and Figures [file 6606056x1.ppt]

## Slide 1
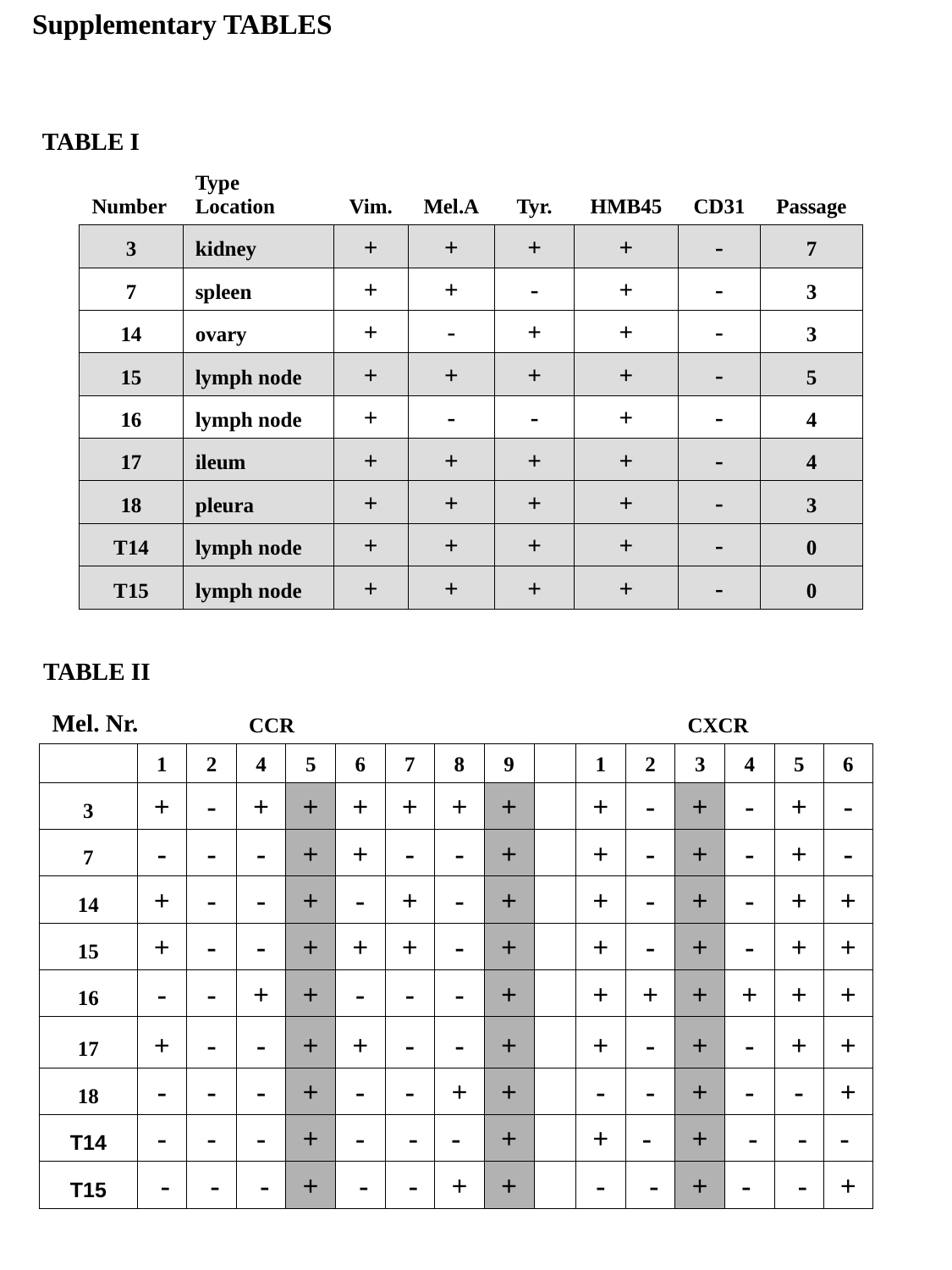

Supplementary TABLES
| | | | | | | | |
| --- | --- | --- | --- | --- | --- | --- | --- |
| Number | Type Location | Vim. | Mel.A | Tyr. | HMB45 | CD31 | Passage |
| 3 | kidney | + | + | + | + | - | 7 |
| 7 | spleen | + | + | - | + | - | 3 |
| 14 | ovary | + | - | + | + | - | 3 |
| 15 | lymph node | + | + | + | + | - | 5 |
| 16 | lymph node | + | - | - | + | - | 4 |
| 17 | ileum | + | + | + | + | - | 4 |
| 18 | pleura | + | + | + | + | - | 3 |
| T14 | lymph node | + | + | + | + | - | 0 |
| T15 | lymph node | + | + | + | + | - | 0 |
TABLE I
| | | | | | | | | | | | | | | | |
| --- | --- | --- | --- | --- | --- | --- | --- | --- | --- | --- | --- | --- | --- | --- | --- |
| Mel. Nr. | | | CCR | | | | | | | | | CXCR | | | |
| | 1 | 2 | 4 | 5 | 6 | 7 | 8 | 9 | | 1 | 2 | 3 | 4 | 5 | 6 |
| 3 | + | - | + | + | + | + | + | + | | + | - | + | - | + | - |
| 7 | - | - | - | + | + | - | - | + | | + | - | + | - | + | - |
| 14 | + | - | - | + | - | + | - | + | | + | - | + | - | + | + |
| 15 | + | - | - | + | + | + | - | + | | + | - | + | - | + | + |
| 16 | - | - | + | + | - | - | - | + | | + | + | + | + | + | + |
| 17 | + | - | - | + | + | - | - | + | | + | - | + | - | + | + |
| 18 | - | - | - | + | - | - | + | + | | - | - | + | - | - | + |
| T14 | - | - | - | + | - | - | - | + | | + | - | + | - | - | - |
| T15 | - | - | - | + | - | - | + | + | | - | - | + | - | - | + |
TABLE II

## Slide 2
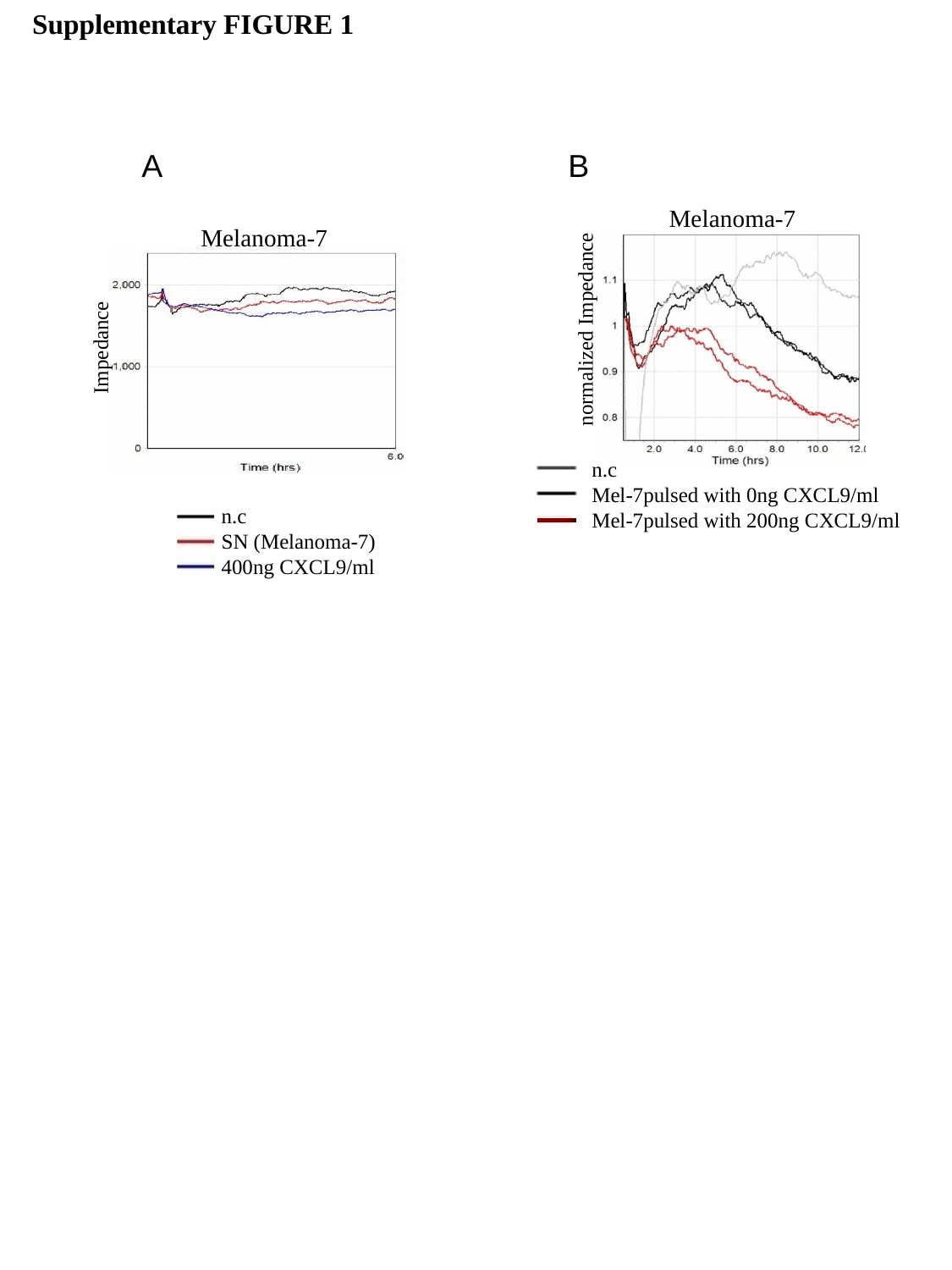

Supplementary FIGURE 1
A
B
Melanoma-7
Melanoma-7
normalized Impedance
Impedance
n.c
Mel-7pulsed with 0ng CXCL9/ml
Mel-7pulsed with 200ng CXCL9/ml
n.c
SN (Melanoma-7)
400ng CXCL9/ml

## Slide 3
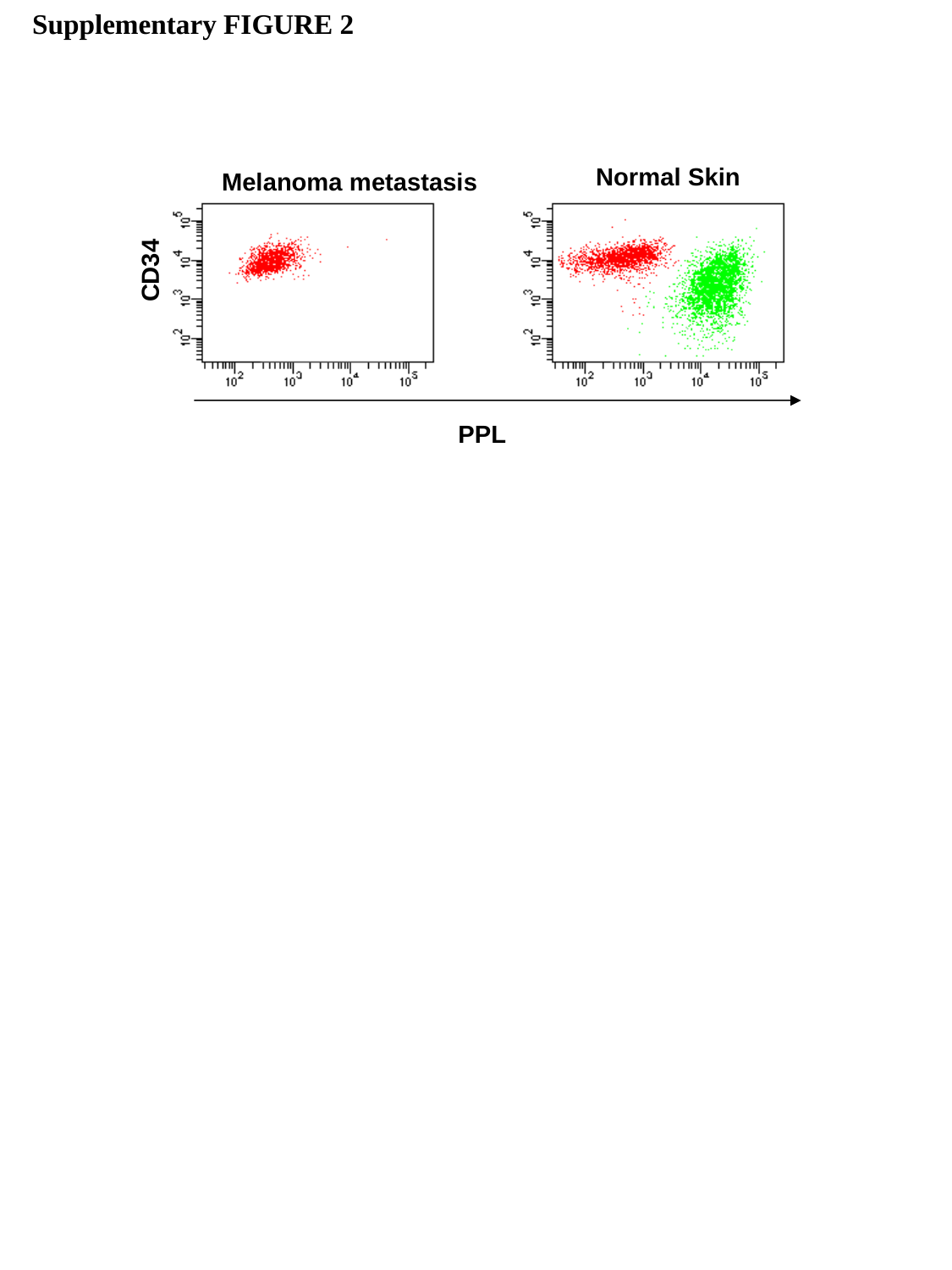

Supplementary FIGURE 2
Normal Skin
Melanoma metastasis
CD34
PPL
