# Supplementary material for: CXCL9 induces chemotaxis, chemorepulsion and endothelial barrier disruption through CXCR3-mediated activation of melanoma cells
Source: Br J Cancer. 2010 Dec 21;104(3):469–79. doi: 10.1038/sj.bjc.6606056 (PMC3049560; doi:10.1038/sj.bjc.6606056)
Supplement: Supplementary Information [file 6606056x2.doc]

**Supplementary Tables**

**Table I** Isolated melanoma cells and marker analysis. Melanoma cells were isolated from metastatic melanoma and either cultivated for several passages (Mel 3-18) or used directly after isolation (T14, T15). The melanoma cells were fixed, permeabilized and analyzed for melanoma markers (Vimentin, Melanin A, Tyrosin, HMB45) and endothelial marker (CD31) as a negative control using FACScan analysis. The percentage of melanoma-marker positive cells was calculated by subtracting the fluorescence of control cells (isotype control) from the cells stained with specific Abs. Samples in which ≥50% of cells displayed a positive signal were indicated as positive (+). Abbreviations: +: positive staining; - negative staining; Vim: Vimentin; Mel.A: Melanin A; Tyr: Tyrosinase; HMB45: human melanosomal antigen; CD31 (PECAM1): Platelet-Endothelial Cell Adhesion Molecule 1; Melanoma cells that stained positive for all melanoma markers are indicated in grey.

**Table II** Expression of Chemokine Receptors CCR and CXCR on melanoma cells. Isolated melanoma cells were fixed, permeabilized and analyzed for chemokine receptor expression by antibody labeling and flow cytometry analysis. The percentage of chemokine receptor positive cells was calculated by subtracting the fluorescence of control cells (isotype control) from the cells stained with specific Abs. Samples in which ≥50% of cells showed a positive signal were indicated as positive (+). Abbreviations: +: positive staining; - negative staining; Chemokine receptors that stained positive in all isolated melanoma cells are indicated in grey.

**Supplementary Figures**

**Figure 1** Increased EC-monolayer disruption during transmigration is caused by CXCL9-activated melanoma cells. **(A**) HUVECs were seeded onto ECIS arrays and allowed to grow to a monolayer before treated with 400 ng/ml soluble CXCL9, Melanoma-7 supernatant (SN) or not treated and incubated for 6 h. Changes in the EC-monolayer due to the CXCL9 or the SN stimulation and EC-monolayer breakdown after melanoma treatment were detected using the impedance measurement (ECIS). (**B**) The experiment was carried out as described above (E) except that not the EC-monolayer but the melanoma cells (Mel-7) were pulsed with 200 ng/ml CXCL9 and washed before they were used to challenge the EC-monolayer.

**Figure 2** Single cell suspensions of subcutaneous melanoma metastases lack podoplanin positive lymphatic endothelial cells. TuECs as well as normal BECs and LECs were identified by a triple labeling protocol. Initially anti CD144-PC5 allowed a clear separation of all ECs from remaining cell types. For the further discrimination between BECs and LECs a combination of anti CD34-PE and anti PDPN-FITC was applied. In normal skin CD144+ cells resolved into two clearly separated populations of CD34+/PDPN- BECs (red) as well as CD34~/PDPN+ LECs (green). In contrast, no PDPN+ population could be detected in CD144+ TuECs from melanoma lesions.

**Materials and Methods**

***Antibodies***

Purified non-labeled mouse monoclonal antibodies were anti-Vimentin and anti-MelanA (Sigma), anti-HMB45 (Dako), anti-tyrosinase (Santa Cruz, CA), anti-CCR3, anti-CCR8 and anti-CCR9 (R&D, Systems, McKinley Place, MN), and **anti-CCR5** (Research Diagnostics). FITC-conjugated mAbs were anti-CCR5 CR3 (R&D Systems). PE-conjugated mAbs were anti-CXCR1, anti CXCR2, anti-CXCR4, anti-CCR4 (Pharmingen, San Diego, CA), anti-CCR7 (BD Biosciences), anti-CCR2, anti-CCR6, anti-CXCR5 and anti-CXCR6 (R&D Systems). Biotinylated anti-CCR1 were obtained from R&D Systems.
